# Peripheral myelin protein-22 (PMP22) modulates alpha 6 integrin expression in the human endometrium

**DOI:** 10.1186/1477-7827-9-56

**Published:** 2011-04-25

**Authors:** Rajiv G Rao, Deepthi Sudhakar, Claire P Hogue, Stephanie Amici, Lynn K Gordon, Jonathan Braun, Lucia Notterpek, Lee Goodglick, Madhuri Wadehra

**Affiliations:** 1 Department of Pathology and Laboratory Medicine, University of California, Los Angeles, California 90095, USA; 2 Department of Neuroscience, McKnight Brain Institute, University of Florida, Gainesville, FL 32610, USA; 3 Department of Ophthalmology, University of California, Los Angeles, California 90095, USA; 4 Jonsson Comprehensive Cancer Center, University of California, Los Angeles, California 90095, USA

## Abstract

**Background:**

PMP22, a member of the GAS3 family of tetraspan proteins, is associated with a variety of neurological diseases. Previous studies have shown that PMP22 is expressed in proliferative endometrium, but its function within this tissue is poorly understood. In this study, we first characterized the expression of PMP22 in the human menstrual cycle and began to characterize its function in the endometrium.

**Methods:**

Using a combination of immunohistochemistry and quantitative PCR, we characterized the expression of PMP22 in both proliferative and secretory endometrium. Differences in PMP22 expression between proliferative and secretory endometrium were determined using a Mann-Whitney U test. In order to investigate the influence of PMP22 on α6 integrin expression, cells were created that ectopically overexpressed PMP22 or expressed a siRNA to inhibit its expression. These cells were analyzed for changes in integrins and binding to extracellular matrices.

**Results:**

In this study, we show that PMP22 expression is higher in proliferative phase than secretory phase. Functionally, we have begun to characterize the functional significance of this expression. Previous studies have suggested a link between PMP22 and α6 integrin, and therefore we asked whether PMP22 could associate or potentially modulate the expression of α6 integrin. Expression of both PMP22 and α6 integrin were detectable in endometrial epithelial and stromal cells, and we show that both proteins can associate and colocalize with each other. To understand if PMP22 directly altered the expression of a6 integrin, we examined cell lines with modulated levels of the protein. Overexpression of PMP22 was sufficient to increase α6 integrin surface expression with a concominant increase in binding to the extracellular matrix laminin, while a reduction in PMP22 suppressed α6 integrin surface expression.

**Conclusion:**

These findings suggest a physiologic role for PMP22 on the expression of α6 integrin. We predict that this may be important for the maintainence of endometrial integrity and to the disease biology associated with altered levels of α6 integrin expression in the endometrium.

## Background

Peripheral myelin protein 22 (PMP22) is a member of the Growth Arrest Specific 3 (GAS3) family of tetraspan proteins. Expression of the PMP22 gene is driven by two alternate promoters P1 and P2 which drive transcription for two transcripts containing different noncoding exons termed 1A and 1B [1]. Although both transcripts translate into identical proteins, the presence of two promoters is thought to confer tissue specific control of expression [2]. Transcripts arising from promoter 1 (termed 1A) have been shown in the peripheral and central nervous systems and are thought to be important for myelin formation [3,4]. Transcripts originating from promoter 2 (termed 1B) have been identified in neuronal and non-neuronal tissue throughout the body [1]. Within non-neuronal tissue, transcripts of PMP22 1B have been identified in the epithelia of the lungs and uterus, the choroid plexus, and the heart [5,6].

Translation of the PMP22 gene gives rise to a 160-amino-acid protein, with four predicted transmembrane domains. The highest expression of PMP22 occurs in Schwann cells, and there, PMP22 localizes strictly with compact myelin [7]. Altered expression of PMP22 has grave consequences as it is associated with certain heritable demyelinating peripheral neuropathies. In particular, elevated expression of PMP22 causes Charcot-Marie-Tooth disease type 1A (CMT1A), an autosomal dominant condition that is characterized by progressive motor and sensory polyneuropathy [8-10]. Haploinsufficiency of PMP22 results in hereditary neuropathy with liability to pressure palsies (HNPP) [11,12].

Outside of its role in myelin formation, studies have implicated PMP22 in a number of cellular roles including adhesion and the regulation of proliferation [13]. In fact, PMP22 was first discovered as a gene upregulated in growth-arrested fibroblasts in culture [14], and since then, PMP22 protein has been shown to help regulate cell spreading and regulate apoptosis in these cells [15]. Its importance in non-neuronal cells was further expanded when it found that in epithelial cells, PMP22 localized within tight junctions and formed complexes with integrins such as α6β4 and with the integral cation channel P2X7 [16-18].

Several studies suggest a complex mechanism for the regulation of PMP22 expression, and recent studies have implicated steroid hormones in its regulation. Studies have shown that both progesterone and glucocorticosteroids act as positive regulators of expression in Schwann cells [19-21], and anti-progesterone therapy has been shown to reduce PMP22 levels, reducing the CMT1A phenotype [22,23]. However, outside of this cell type, limited information is available as to hormonal control of PMP22 expression. PMP22 has been observed in the uterus, with high PMP22 mRNA levels in proliferative stroma [24], but no studies have examined PMP22 expression in epithelial cells of the endometrium or throughout the estrous cycle.

In this study, we characterize the expression of human PMP22 during the proliferative and secretory phases of the female menstrual cycle. As previous studies have suggested that PMP22 associates with integrins, we generated human endometrial cell lines with varying levels of PMP22 expression and characterized their integrin profiles. We report for the first time, the expression of PMP22 protein in the human endometrium, with greater expression in the proliferative phase as compared to the secretory phase. Furthermore, we show that PMP22 colocalizes α6 integrin both in vitro and in normal human tissue samples. The dichotomy of PMP22 and α6 integrin expression in the female menstrual cycle suggests roles for the both proteins in adhesion and state of endometrial differentiation.

## Methods

### Cell lines

HEC-1A, Human endometrial adenocarcinoma cells (HTB112, ATCC, Manassas, VA) were cultured in McCoys media, supplemented with 10% fetal calf serum, 1% L-Glutamine, 1% penicillin/streptomycin at 37°C in a humidified 5% CO_2 _incubator. Cells were passaged every 3-4 days. HEC-1A cells were stably transfected with expression plasmids for a control eGFP, or a human PMP22 open reading frame tagged with a myc-epitope in the second extracellular loop using FuGENE 6 (Roche Molecular Biochemicals) per the manufacturer's instructions. These expression vectors have been previously described [17,25]. Stable cellular clones were selected using Geneticin (800 ug/ml; Invitrogen, Carlsbad, CA) and referred to as HEC-1A/Vector (empty vector control), and HEC-1A/PMP22 (PMP22 overexpression). In some experiments, PMP22 levels were decreased by transiently transfecting HEC-1A cells with 75 picomoles PMP22 siRNA duplexes (SMARTpool L-010616-00-0005; ThermoScientific, Boulder, CO) and a lipophilic transfection reagent (Lipofectamine 2000; Invitrogen, Carlsbad, California) for 48 hours. As a negative control, cells were transfected with 75 picomoles scrambled control siRNA (D-001206-13-05; ThermoScientific). Cells were harvested for Western blot, flow cytometry, immunofluorescence or immunoprecipitations 36-48 hours post-transfection, as detailed below.

### Human tissue

Retrospective human tissue was obtained from the UCLA Department of Pathology and Laboratory Medicine Tissue Procurement Core Facility (TPCL) under an exemption from the Institutional Review Board. To validate PMP22 expression in human endometrium, we tested total PMP22 expression in whole tissue fragments of proliferative endometrium (*n *= 6) and secretory endometrium (*n *= 6). As controls, we also obtained normal frozen tonsil and lung. Tissue samples were collected and frozen in the -80 C. These tissues were prepared in the hospital biorepository, and clinical annotation is available though a database. Cases were classified and selected based on a normal diagnosis using the Anatomic Pathology system, and no information regulated by HIPPA was included in the study, which qualifies for the status of NIH Exemption #4.

### RT-PCR

All tissue was homogenized and RNA isolated using the Qiagen RNAeasy kit per manufacturer's instructions. The Qiagen OneStep RT-PCR kit (Valencia, CA) was employed using human PMP22 exon 1A or 1B [4] or GAPDH [26]-specific primers using 1 μg RNA.

PMP22 1A: 5'- TTACAGGGAGCACCACCA-3'

PMP221B: 5'-CCACGCACCCGAGTTTGT-3'

PMP22 R: 5'-ATCATGGTGGCCTGGACA-3'

The RT-PCR conditions were reverse transcribed at 50°C for 30 min followed by PCR (25-40 cycles) at 95°C (30 s), 55°C (30 s), and 72°C (1 min).

In order to quantititate mRNA levels, the Qiagen QuantiTect SYBR Green RT-PCR Kit was utilized. Primers were synthesized (Real Time Primers, Elkins Park, PA) which could recognize both transcripts of PMP22. The 25 μl PCR reaction included 12.5 μl 2 × RT-PCR Master Mix, 0.5 μmol/L forward primer, and 0.5 μmol/L reverse primer. The reactions were incubated in a 96-well plate and reverse transcribed and amplied on the Applied Bio Systems7500 Fast System (Carlsbad, CA) using the following primers:

PMP22 F: 5'- GTATCATCGTCCACGTC-3'

PMP22 R: 5'-GGCAGAAGAACAGGAACAGA-3'

The 2^-⊿⊿Ct ^method for relative quantification of gene expression was used to determine PMP22 expression levels [27]. Each sample was analyzed in triplicate, and the housekeeping gene GAPDH was used to normalize expression as previously described [28]. Differences between PMP22 expression in proliferative and secretory endometrium was determined using Mann-Whitney U test.

### Western blot analysis

Tissue or cell lines were lysed in Laemmli buffer as previously described [29] and analyzed for PMP22 expression by SDS-PAGE Western blot analysis. Approximately 25 μg of protein were loaded per lane. In order to detect PMP22 expression, samples were treated for 2 h at 37°C with PNGase (New England Biolabs, Beverly, MA) to remove N-linked glycans [30]. Proteins were then separated by SDS-PAGE and transferred to a nitrocellulose membrane (Invitrogen). Following transfer, membranes were stained with Ponceau S (Sigma-Alrich, St. Louis, MO) to validate the efficiency of transfer. A solution of 10% nonfat milk in Tris buffered saline containing 0.1% Tween-20 was used to block non-specific binding. Membranes were probed with rabbit anti-PMP22 antisera [6] or with anti-β-actin antibodies (Sigma-Aldrich). Protein bands were visualized using a horseradish peroxidase-labeled secondary antibody (Southern Biotechnology Associates, Birmingham, AL) followed by chemiluminescence (ECL; Amersham Biosciences). Band intensities were quantified using the NIH program Image J. Blots were digitized using a flatbed scanner and the band density measured using Image J. The relative intensity of PMP22 in the three cell lines was calculated by dividing the volume intensities over that of the β-actin control. Experiments were repeated three times and averaged.

### Immunoprecipitations

Cells were washed in PBS and solubilized in lysis buffer for 30 min at 4°C. (1% Nonidet P-40 containing 2 mM phenylmethylsulfonyl fluoride, 10 ug/ml aprotinin, 2 ug/ml pepstatin, 10 mM iodoacetamide, 0.1 mM EDTA, 10 mM HEPES, and 10 mM KCl). Lysates were sonicated for 15 seconds and pre-cleared by incubation with protein A-agarose beads (Santa Cruz Biotechnology, Inc., Santa Cruz, CA). Pre-cleared lysates were incubated overnight with Protein-A beads bound to either anti-PMP22 rabbit polyclonal antisera (Sigma-Aldrich), anti-α6 integrin rabbit polyclonal antibody (Santa Cruz Biotechnology), or control rabbit sera. The beads were washed once in lysis buffer and 50 mM Tris buffer to neutralize salt. Immune complexes were eluted from the beads with Laemmli buffer. Cell lysates were subsequently analyzed by Western blots.

### Immunofluorescence

HEC-1A cells were plated onto glass coverslips (Fisher Scientific, Pittsburgh, PA) and incubated for 24 h at 37°C. Cells were fixed in cold methanol, blocked in 1% normal goat serum and washed in PBS with 0.01% Triton X-100. Cells were subsequently incubated at 4°C with PMP22 rabbit antisera (1:100; Sigma, CA), anti-α6 integrin rat antibody (1:100; BD Biosciences) in a humidified chamber. Cells were rinsed with PBS with 0.01% Triton X-100, then incubated for 1 hour at RT with Alexa Fluro 488-conjugated goat anti-rabbit IgG (1:50), or fluorescein isothiocyanate (FITC)-conjugated anti-rat IgG2a (1:450; eBiosciences). Negative controls included incubation with secondary antibody alone. Cells were briefly washed in PBS with 0.01% Triton X-100, double deionized H_2_O, and mounted onto slides in a 3.5% n-propyl gallate-glycerol solution. Mounted coverslips were analyzed for PMP22 and α6 integrin localization using confocal microscopy.

### Flow cytometry

The membrane expression of α6 integrin, α2 integrin, and αvβ3 integrin were assessed by flow cytometry. Cells were fixed in 2% paraformaldehyde (w/v) in PBS for 20 min on ice. Cells were pelleted and resuspended in PBS with 2% fetal calf serum (FCS). Cells were incubated with primary antibody (1:100) and incubated at RT for 20 min. Cells were then incubated with red-phycoerythrin-conjugated anti-rat Ig-κ light chain antibody or red-phycoerythrin-conjugated anti-mouse Ig-κ light chain antibody for 20 min (0.25 ug/ 10^6 ^cells; BD Biosciences). Negative control cells were treated similarly, but without primary antibody. After two consecutive washes with PBS, cells were resuspended in PBS and analyzed with a FACScan flow cytometer (BD Biosciences). Integrin expression levels were calculated as mean fluorescent intensity (MFI) using CellQuest software. Experiments were performed in triplicate.

### Immunohistochemistry

Retrospective formalin fixed, paraffin embedded normal human proliferative or secretory endometrial samples were obtained from the TPCL at UCLA. Whole tissue sections of proliferative endometrium (*n = 6*) or secretory endometrium (*n = 6*) were analyzed by immunohistochemistry for PMP22 or α6 integrin expression. Briefly, paraffin-embedded human tissue samples were deparaffinized, blocked for endogenous peroxidase activity with 3% hydrogen peroxide, and heated at 95°C for 20 min with citrate buffer (19). The tissue samples were blocked with normal goat serum in TBS with Tween for 10 min and stained with rabbit-anti-PMP22 polyclonal antibody (1:100; Sigma-Aldrich) or anti-α6 integrin rabbit antibody (1:100; Santa Cruz Biotechnology Inc., Santa Cruz CA) overnight in a humidified chamber. Slides were developed by incubation with a biotinylated secondary antibody from the Vectastain Elite ABC kit (Vector Laboratories) according to the manufacturer's protocol, followed by a diaminobenzidine tetrahydrochloride (DAB) substrate solution (Pierce, Rockford, IL). Nuclei were counterstained with hematoxylin. Slides were analyzed for PMP22 and α6 integrin expression by microscopy.

To detail the staining of PMP22 and α6 integrin in proliferative and secretory endometrium, a semi-quantitative analysis was performed. Each section stained was assessed by considering the staining intensity (0 = below the level of detection, 1, weak; 2, moderate; and 3, strong) and the percentage of cells staining at each intensity level (0-100%). For each tissue, an integrated value of intensity combined with frequency was derived using the formula: [(3x) + (2y) + (1z)] / 100 where x, y, and z are % staining at intensity 3, 2, and 1, respectively. Differences in expression between proliferative and secretory endometrium were determined using a Mann-Whitney test, where p < 0.05 was considered significant.

### Adhesion assays

A standard static cell adhesion assay (15-20 min) was performed as previously described [30]. Briefly, 96-well plates were precoated for 2 hours at 37°C with the ECM substrates laminin, fibronectin, poly-D-lysine (positive control; Roche Molecular Biochemicals; 5-10 μg/ml), or 1% fatty acid-free bovine serum albumin (negative control; Sigma-Aldrich). Cells (7 × 10^4^) were plated onto the ECM in serum-free conditions and incubated at 37°C for 30 min. Unbound cells were washed away. Bound cells were fixed, stained with toluidine blue and then lysed using 2% SDS (Biowhittaker, Walkersville, MD). The resultant soluble toluidine blue was quantitated by measuring the absorbance at 595 nm. Binding to each ECM was performed in triplicate. Each experiment was repeated at least three times. An unpaired Student's *t *test was used to confirm significance between HEC-1A/PMP22, HEC-1A/Vector, HEC-1A/scrambled siRNA control, and HEC-1A/PMP22 siRNA cells.

In antibody blocking experiments, cells were preincubated with various dilutions of anti-α_6 _integrin [31] or anti-α_2 _integrin [32] function blocking antibodies (BD Biosciences) for 60 min at 4°C. Cells (5-7 × 10^4^) were plated in triplicate into a 96-well plate precoated with laminin or poly-D-lysine and allowed to adhere for 30 min. Unbound cells were washed away, and bound cells were quantitated as described above. Each experiment was repeated three times.

## Results

### Characterization of PMP22 expression

**Figure 1:**
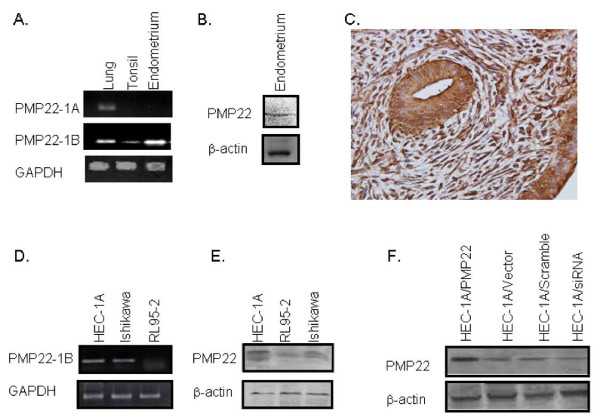
**PMP22 is expressed in the endometrium**. (A). Frozen adult human tissues were obtained, and total RNA isolated. PMP22 exon 1B mRNA was expressed in endometrium, lung, and tonsil tissue while expression of PMP22 exon 1B mRNA was only observed in the lung. GAPDH mRNA was used as the loading control. (B) Tissue lysates were prepared to determine PMP22 protein expression in the endometrium. PMP22 protein was detectable in proliferative endometrium, and a representative sample is depicted. N = 6. (C) PMP22 expression was confirmed in human proliferative endometrium using immunohistochemistry, and a representative sample is displayed. N = 6. Magnification = 400 ×. (D) PMP22 mRNA transcripts were detected in HEC-1A, Ishikawa, and RL95-2 endometrial cell lines using semi-quantitative RT-PCR. GAPDH was used as a loading control. (E) PMP22 protein levels in the three immortalized endometrial cell lines were detected using PMP22 antisera. β-actin was used as a loading control. (E) To study PMP22 in HEC-1A epithelial cells, its expression was modulated through ectopic overexpression, or by inhibition using a PMP22 specific siRNA. Vector and scrambled siRNA controls are included for endogenous expression. Expression of PMP22 was confirmed by western blot analysis, with β-actin levels serving as a loading control.

Previous studies have shown that PMP22 is expressed in proliferative endometrium [24]. In order to confirm the expression of PMP22 within the endometrium, we first confirmed the mRNA and protein expression of PMP22 in human endometrium. PMP22 expression has been shown to be regulated by two alternatively used promoters which are located upstream of two distinct 5' non-coding exons (exons 1A and 1B), but both transcripts encode for a single protein [3,33]. To characterize both transcripts in the human endometrium, RT-PCR was performed on select human tissue. In the endometrium, PMP22 exon 1B mRNA expression was detectable, and this transcript was also found in low levels in the tonsil and in the lung (Figure 1A). Interestingly, PMP22 exon 1A mRNA was only detectable in the lung. We next confirmed the protein expression of PMP22 using western blot and immunohistochemical analysis. PMP22 protein expression was observed in a representative human proliferative endometrial sample (Figure 1B) and within proliferative endometrial epithelial and stromal cells (Figure 1C).

In order to investigate the function of PMP22 in endometrial epithelial cells, we monitored PMP22 expression in a panel of endometrial cancer cell lines. Similar to the mRNA profile observed above, both HEC-1A and Ishikawa endometrial cell lines were positive for exon 1B expression (Figure 1D). The expression of PMP22 mRNA was below detection in RL95-2 cells. To translate PMP22 expression in these cells, we characterized the relative PMP22 protein expression by western blot analysis (Figure 1E). PMP22 was expressed in all three cell lines, with the highest expression observed in HEC-1A cells. Moreover, very low levels of PMP22 were observed in the RL95-2 cells.

To investigate the role of PMP22 in endometrial epithelial cells, we created a panel of HEC-1A cells with modulated PMP22 expression levels (Figure 1F). HEC-1A/PMP22 overexpressed myc-tagged human PMP22, HEC-1A/Vector expressed a vector control and baseline levels of endogenous PMP22, and HEC-1A/siRNA expressed PMP22-targeted siRNA and thus, reduced levels of PMP22. In some experiments we also utilized a scrambled siRNA control, which phenotypically resembled the vector control. Based on semi-quantitative analysis, we obtained a 3 ± 0.2-fold increase in the levels of PMP22 in the overexpressing cells. In comparison, the siRNA reduced endogenous PMP2 by 50 ± 5% (Figure 1F). No difference in PMP22 expression was observed between the vector control and scrambled siRNA control.

### Changes in PMP22 expression regulates α6 integrin expression

**Figure 2:**
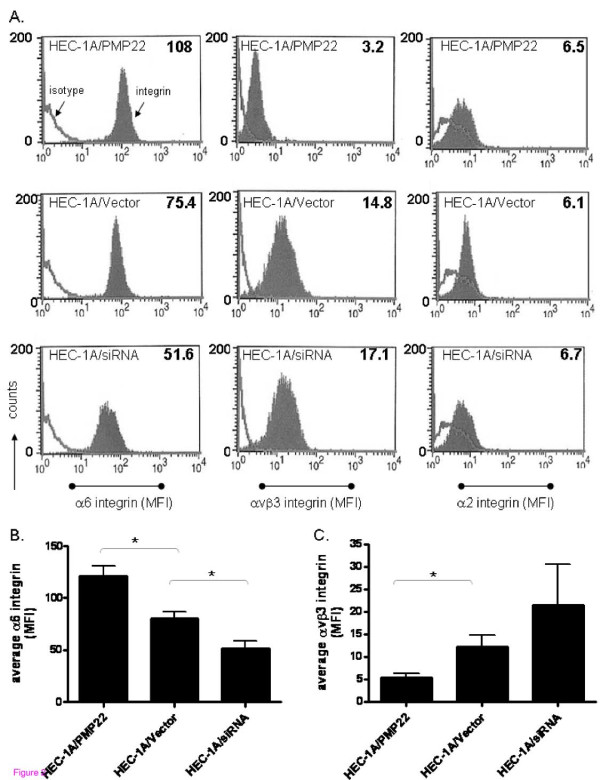
**PMP22 expression differentially regulates select integrin pairs**. The surface expression of α6, αvβ3, or α2 integrin in HEC-1A/PMP22, HEC-1A/Vector, or HEC-1A/PMP22 siRNA cells was determined using flow cytometry. The MFI was quantitated and tabulated in the top corner of each histogram. Upregulation of PMP22 promoted the surface expression of α6 integrin, while slightly reducing αvβ3 integrin. Reciprocal effects were seen with the PMP22 specific siRNA. In contrast, no change in α2 integrin expression was observed when PMP22 expression was modulated. The experiment was repeated three times, and a representative experiment is shown. Statistical differences between α6 integrin (B) and αvβ3 (C) expression were determined between the three cell lines. *, *p *< 0.05; Student's unpaired *t *test.

We next investigated whether PMP22 expression regulated the surface expression of specific integrin subunits. Flow cytometry was used to study select integrin expression in HEC-1A/PMP22, HEC-1A/Vector, HEC-1A/scrambled control, and HEC-1A/siRNA cells. Overexpression of PMP22 resulted in increased expression of α6 integrin compared to HEC-1A/Vector control cells (Figure 2). Interestingly, a reduction of PMP22 expression with siRNA also reduced the expression of α6 integrin relative to vector control cells. This affect was exclusive to α6 integrin because overexpression of PMP22 did not alter the expression pattern of the integrin α2 subunit (Figure 2). Moreover, HEC-1A/PMP22 inversely modulated the expression of αvβ3 integrin. High levels of PMP22 slightly reduced the expression of αvβ3 integrin, while transfection with PMP22 siRNA slightly increased the expression of this integrin pair (Figure 2). No differences between the vector control and scrambled siRNA controls were observed (data not shown).

### Changes in PMP22 expression influence cell adhesion to extracellular matrix proteins

**Figure 3:**
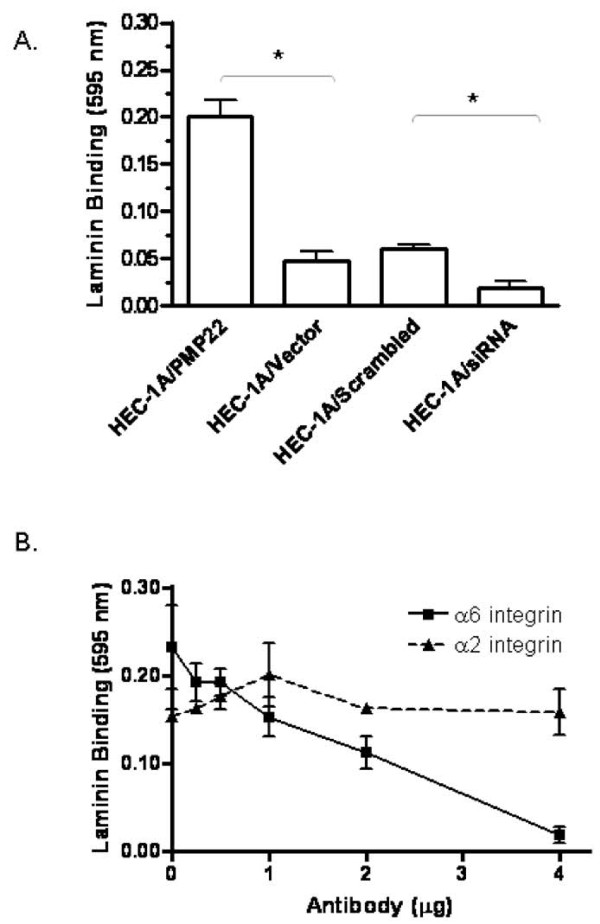
**PMP22 expression increased binding on laminin**. (A) 5 × 10^4 ^cells (HEC-1A/PMP22, HEC-1A/Vector, HEC-1A/scrambled siRNA control, and HEC-1A/PMP22 siRNA) were incubated in serum-free media at 37°C for 30 min in wells coated with different extracellular matrices. Absorbance was measured at 595 nm. Values are represented as the mean ± SEM. for triplicate wells (*p < 0.05; Student's unpaired *t *test). A representative experiment is shown; three independent experiments yielded similar results. (B) In order to confirm that laminin binding was α6 integrin dependent, HEC-1A/PMP22 cells were incubated with indicated concentrations of α6 or α2 (control) integrin antibodies. Samples were then plated on laminin for 30 min, and analyzed as above.

One important function of integrins is to bind to extracellular matrix proteins. To translate the functional effect of changes in PMP22 expression levels on α6 integrin ligand specificity, HEC-1A/PMP22, HEC-1A/PMP22 siRNA, HEC-1A/scrambled control, and HEC-1A/Vector control cells were tested for their binding capacity to the extracellular matrix laminin. Overexpression of PMP22 resulted in a significant increase in binding to laminin while in contrast, PMP22 siRNA reduced laminin binding (Figure 3A). To confirm that laminin binding was α6 integrin-dependent, HEC-1A cells were incubated for 30 min with increasing concentrations of either α6 or α2 integrin blocking antibodies (Figure 3B). PMP22 binding to laminin was specifically disrupted when incubated with α6 integrin antibodies, but was not affected by α2 integrin antibodies. These results strongly suggested that the tetraspan protein PMP22 may help regulate α6 integrin-mediated adhesion.

### PMP22 and α6 integrin coimmunoprecipitate

**Figure 4:**
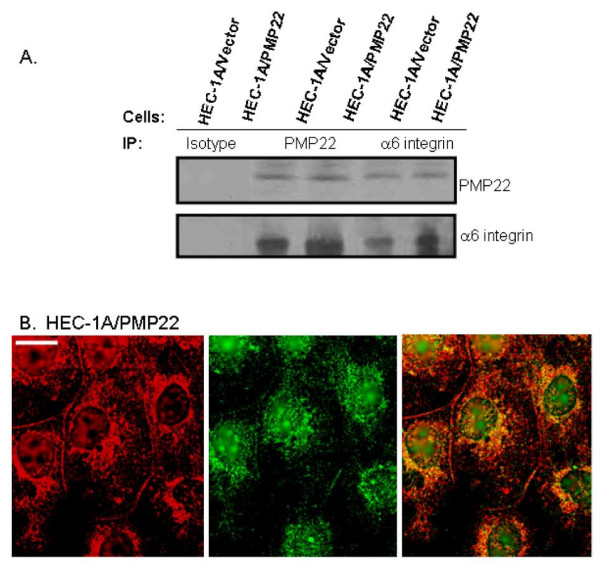
**PMP22 and α6 integrin associate with each other**. HEC-1A/Vector or HEC-1A/PMP22 cells were lysed in 1% NP-40 and incubated with PMP22 or α6 integrin antisera. Immunoprecipations were assessed by SDS-PAGE analysis. (A) α6 integrin and PMP22 co-immunoprecipitate in HEC-1A/Vector and HEC-1A/PMP22 cells when using either PMP22 or the α6 integrin antibody. Normal rabbit sera were used to assess nonspecific pull-down of proteins. (B) Confocal images of HEC-1A/PMP22 cells after co-immunolabeleing with anti-PMP22 (Rhodamine) and anti-α6 integrin (clone G0H3; FITC) antibodies. Co-localization of PMP22 protein (Rhodamine) with α6 integrin (FITC) was observed in both intracellular and plasma membrane compartments. Scale bar, 10 μM. Insets, 50 μM.

As the flow cytometry data suggested that expression of α6 integrin was altered by changes in PMP22 expression, we started to characterize this correlation. Initially, we determined if PMP22 and α6 integrin could associate. Immunoprecipitations were performed in HEC-1A/Vector and HEC-1A/PMP22 cells. In HEC-1A/Vector and HEC-1A/PMP22 overexpressing cells, use of an anti-PMP22 antibody pulled down both α6 integrin and PMP22 (Figure 4A). Concordantly, pull-down with anti-α6 integrin antibody resulted in detectable PMP22 and α6 integrin (Figure 4A).

We also utilized confocal microscopy to examine the co-localization of PMP22 and α6 integrin. HEC-1A/PMP22 and HEC-1A/Vector cells were stained with anti-PMP22 (red) and anti-α6 integrin antibody (green). In HEC-1A/Vector cells, PMP22 and α6 integrin exhibited a similar pattern of expression (data not shown). Specifically, both proteins were largely retained in intracellular compartments, although some protein could be detected on the plasma membrane. Upon overexpression of PMP22 in HEC-1A/PMP22 cells, significant levels of both a6 integrin and PMP22 were visualized (Figure 4C). Both proteins colocalized on the plasma membrane as well as in intracellular compartments. Thus, overexpression of PMP22 results in increased expression of α6 integrin at the plasma membrane, further supporting the association of PMP22 and α6 integrin.

### PMP22 is differentially expressed in the menstrual cycle

**Figure 5:**
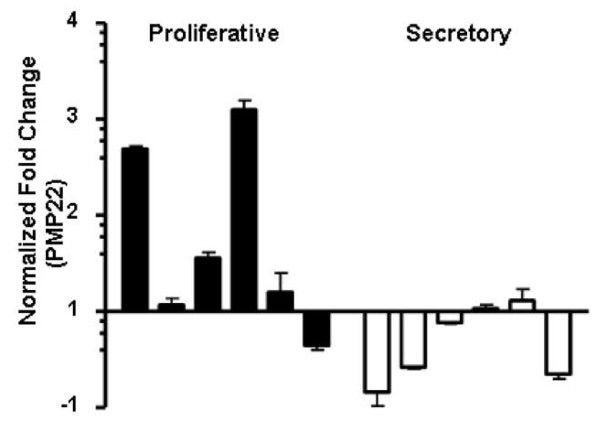
**PMP22 mRNA levels inversely correlate in proliferative and secretory phase samples**. Normal proliferative (N = 6) and secretory (N = 6) tissue was analyzed for PMP22 expression levels. GAPDH was used to normalize between samples. Significant differences exist in PMP22 expression between normal proliferative and secretory tissues. *p = 0.02.

Several studies have suggested that PMP22 is regulated by steroid hormones [20,34]. To examine its expression in the human menstrual cycle, PMP22 mRNA levels in proliferative and secretory endometrium were quantitated using real-time PCR. As shown in Figure 5, PMP22 mRNA levels were significantly down-regulated in secretory endometrium (n = 6) when compared to those in the proliferative phase (*p *= 0.02, Mann Whitney U test). On average, the PMP22 mean expression level (1.7 ± 0.4) was 2.5 fold higher than the mean expression of the secretory samples (0.7 ± 0.2). These results confirm that PMP22 expression is expressed in the endometrium and indicate that PMP22 expression is hormonally regulated in this tissue.

### Localization of PMP22 and α6 integrin in human endometrium

**Figure 6:**
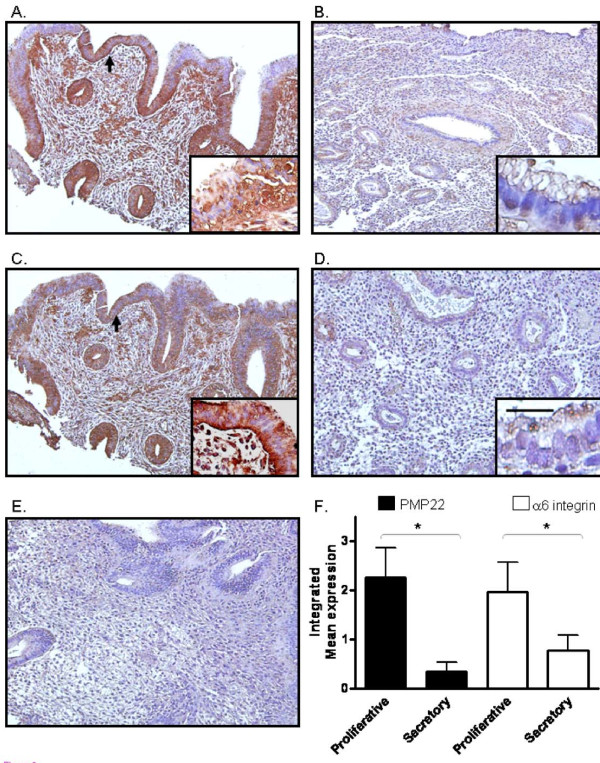
**PMP22 and α6 integrin share a similar pattern of expression in human endometrium**. (A, C) Normal human proliferative endometrium was stained by immunohistochemistry for either PMP22 or α6 integrin, respectively. Bound antibodies were visualized using DAB, and nuclei counterstained using hemotoxylin. Magnification: 100X. Insets depict a 400X enlargement of glandular epithelium. (B, D) Normal human secretory endometrium was stained by immunohistochemistry for either PMP22 or α6 integrin as above. Magnification: 100X. Insets depict a 400X enlargement of glandular epithelium. (E) Normal human proliferative endometrium was stained as above using non-specific control rabbit sera. (F) The mean integrated intensity of PMP22 and α6 integrin protein expression was quantitated for each sample; N = 6 per group. The error bars represent the standard error of the mean. *p< 0.05.

Endometrial sections were stained to characterize the expression of PMP22 in proliferative and secretory phases. PMP22 expression exhibited a phase specific expression pattern. High expression of PMP22 was observed in the proliferative phase (Figure 6A), and it was markedly reduced in the secretory phase (Figure 6B). Within proliferative endometrium, intense PMP22 staining was observed in both glandular and luminal epithelium as well as in the stroma (Figure 6A). Moreover, PMP22 localized with the basolateral interface within glandular epithelium (arrow).

To validate the co-expression of PMP22 and α6 integrin in the endometrium, human endometrial tissues were also probed for α6 integrin expression. Concordantly, during the proliferative phase, expression of α6 integrin was detectable in the glandular epithelium, luminal epithelium, and in the surrounding stromal cells (Figure 6C), and its expression was reduced in secretory endometrium (Figure 6D). Moreover, although α6 integrin showed some staining on the apical surface, there was also pronounced expression on the basolateral side during the proliferative phase (Figure 6A and 6C, arrows). The specificity of staining for both PMP22 and α6 integrin was confirmed using control rabbit sera on a section of secretory endometrium (Figure 6E). To quantitate the observed differences of PMP22 and α6 integrin expression in the proliferative and secretory phases, 12 normal endometrial samples were scored for intensity and the percentage of cells stained. Both PMP22 and α6 integrin were significantly higher in proliferative compared to secretory endometrium (Figure 6F).

## Discussion

Limited data are available on the role of PMP22 in epithelial cell models and within the endometrium. In this study, we confirm the expression of PMP22 in the endometrium, and show that PMP22 is expressed in human glandular, luminal, and stromal cells of the endometrium. Within the menstrual cycle, PMP22 expression is greater during the proliferative phase as compared to the secretory phase, and this expression correlates with similar changes in α6 integrin expression. Mechanistically, both proteins associate in the same complex as determined by immunoprecipation and colocalize by immunoflorescence. Furthermore, elevation of PMP22 expression in HEC-1A cells increases the expression of α6 integrin; conversely, reduction of PMP22 expression also reduces the levels of α6 integrin. Importantly, these changes in integrin surface expression are functionally relevant as corresponding changes in the level of cell adhesion to the extracellular matrix protein laminin are observed.

We propose that PMP22 expression and association with α6 integrin may be important to maintain endometrial integrity. α6 integrins have an important function in not only the attachment of cells to the extracellular matrix, but also in the induction of cell migration and invasion [35,36]. Within the endometrium, α6 integrin has been shown to dimerize with both β1 and β4 integrin. Although it is not known if PMP22 preferentially associates with α6β1 or α6β4 integrins, the localization of PMP22 on the basal surface of endometrial cells suggests an association with α6β4 integrin [35]. This correlation is supported by recent studies in clone A colonic carcinoma cells showing that PMP22 forms a complex with α6β4 integrin [18]. Thus, it is possible that PMP22 helps stabilize or anchor α6β4 integrins on endometrial epithelial cells to the underlying basement membrane, which is known to be comprised of laminin and collagen [37,38] Intriguingly, laminin expression is rich during the proliferative phase and reduced during the secretory phase [38]. Additional experiments will be needed to confirm the association and the specificity of PMP22 and α6 integrin within endometrial cells.

PMP22 belongs to the GAS3 family of tetraspan proteins, and each family member shares similar primary and secondary structures, with four relatively conserved transmembrane regions and two significant extracellular loops [39,40]. Interestingly, another GAS3 family member EMP2 has been detected in the endometrium [25,28,41]. With regard to EMP2 and PMP22, a unique regulatory phenotype has been observed within endometrial cells. Whereas PMP22 is expressed predominantly in proliferative endometrium, EMP2 expression is greater in the secretory phase [28]. Moreover, changes in PMP22 and EMP2 expression appear to modulate different integrin pairs. Overexpression of EMP2 results in the increase of αvβ3-integrin whereas elevation of PMP22 selectively increases the expression of α6 integrin [25]. It is thus intriguing to speculate that this divergent affect on integrin expression may be translated to distinct effects on cellular function.

Importantly, our study confirms that steroid hormones can regulate the expression of PMP22 and concordantly the α6 integrin in non-neuronal tissue. Several studies have shown that in Schwann cells, PMP22 can be hormonally activated. Both glucocortosteroids and 5*α*-androstan-3*α*, 17*β*-diol (3*α*-diol), a metabolite of dihydrotestosteone, has been shown to transiently upregulate PMP22 1A mRNA levels [20,21]. Moreover, anti-progesterone treatments have been shown to improve the phenotype for CMT1A through the specific reduction of PMP22 in Schwann cells [22,23]. Our results suggest that the regulation of PMP22 1A and 1B is different. Given its high expression in the proliferative phase, we would predict that estrogen may upregulate the expression of the PMP22 1B transcript in the endometrium. Additional experiments will be needed to characterize differences in the promoters of each transcript.

Finally, our data suggest that elevated or reduced PMP22 levels could result in an altered repertoire of integrin isoform expression and perhaps specifically with α6 integrin. As dysregulation of α6 integrin has been implicated in disease biology of the endometrium, PMP22 may be an underappreciated potential target for diseases such as infertility, endometriosis, and endometrial cancer [35,42,43].

## Abbreviations

PMP22: peripheral myelin protein-22; EMP2: epithelial membrane protein 2; GAS3: growth arrest specific 3.

## Competing interests

The authors declare that they have no competing interests.

## Authors' contributions

RGR and DS carried out the immunoprecipitation and microscopy experiments. CPH performed the real time PCR and immunohistochemistry experiments. SA and LN participated in the design of the study. LG and MW conceived of the study, and JB and LKG participated in its design and coordination. All authors read and approved the final manuscript.
